# Diagnostic comparison of vibration-controlled transient elastography and MRI techniques in overweight and obese patients with NAFLD

**DOI:** 10.1038/s41598-022-25843-6

**Published:** 2022-12-19

**Authors:** Asako Nogami, Masato Yoneda, Michihiro Iwaki, Takashi Kobayashi, Takaomi Kessoku, Yasushi Honda, Yuji Ogawa, Kento Imajo, Takuma Higurashi, Kunihiro Hosono, Hiroyuki Kirikoshi, Satoru Saito, Atsushi Nakajima

**Affiliations:** 1 Department of Gastroenterology and Hepatology, Yokohama City University Graduate School of Medicine, 3-9 Fukuura, Kanazawaku, Yokohama, 236-0004 Japan; 2 Department of Palliative Care, International University of Health and Welfare Narita Hospital, 852 Hatakeda Narita, Chiba, 286-8520 Japan; 3 Department of Gastroenterology, National Hospital Organization Yokohama Medical Center, 3-60-2 Harajuku, Totsukaku, Yokohama, 245-8575 Japan; 4 Department of Gastroenterology and Endoscopy, Shinyurigaoka General Hospital, 255 Furusawatsuko, Asoku, Kawasaki, 215-0026 Japan; 5 Clinical Laboratory Department, Yokohama City University Hospital, 3-9 Fukuura, Kanazawaku, Yokohama, 236-0004 Japan

**Keywords:** Non-alcoholic fatty liver disease, Non-alcoholic steatohepatitis

## Abstract

Non-invasive imaging techniques have greatly advanced the assessment of liver fibrosis and steatosis but are not fully evaluated in overweight patients. We evaluated the diagnostic performance of vibration-controlled transient elastography (VCTE) and magnetic resonance elastography (MRE) to assess fibrosis and controlled attenuation parameter (CAP) and MR imaging (MRI)-proton density fat fraction (MRI-PDFF) to assess steatosis in overweight and obese patients with non-alcoholic fatty liver disease (NAFLD). We included 163 biopsy-proven patients with NAFLD who underwent VCTE, MRE/MRI-PDFF, and liver biopsy (years 2014–2020) who were classified according to their body mass index (BMI) as normal (BMI < 25 kg/m^2^, n = 38), overweight (25 ≤ BMI < 30 kg/m^2^, n = 68), and obese (BMI ≥ 30 kg/m^2^, n = 57). VCTE and MRE detected fibrosis of stages ≥ 2, ≥ 3, and 4 with an area under the receiver operating curve (AUROC) of 0.83–0.94 (VCTE) and 0.85–0.95 (MRE) in all groups, without considerable differences. MRI-PDFF detected steatosis of grades ≥ 2 and 3 with high AUROC in all groups (0.81–1.00). CAP’s diagnostic ability (0.63–0.95) was lower than that of MRI-PDFF and decreased with increasing BMI compared to MRI-PDFF. VCTE and MRE similarly accurately assess fibrosis, although MRI-PDFF is more accurate than CAP in detecting steatosis in overweight and obese patients with NAFLD.

## Introduction

The number of patients diagnosed with non-alcoholic fatty liver disease (NAFLD) is rising worldwide^1^. Obesity is a key risk factor for NAFLD development, and it is estimated that 90% of obese people suffer from NAFLD^2^. Obesity increases the risk of NAFLD by almost fivefold^3^ and accelerates its progression to advanced fibrosis, cirrhosis, and non-alcoholic steatohepatitis (NASH)^4^. Liver biopsy is currently the gold standard method for diagnosing liver fibrosis, steatosis, inflammation, and hepatocyte ballooning in NAFLD. However, there are limitations to consider when performing liver biopsy, including the invasiveness of the procedure, and the risk of sampling errors. Therefore, elastography was developed as an alternative, non-invasive technique. The accurate assessment of liver fibrosis is essential for patients with NAFLD as this is the most important pathological determinant of prognosis^5–7^.

The estimated prevalence of NAFLD among Asians is 27.4% (95% confidence interval CI 23.3–31.9%)^8^, and it is reported that individuals with overweight and obese have increased risk of NAFLD and 50.7% of them has NAFLD^9^. It remains unclear whether elastography has sufficient diagnostic ability for overweight and obese patients. In the evaluation of NAFLD, magnetic resonance imaging (MRI)-based techniques have been reported to have a better diagnostic performance than vibration-controlled transient elastography (VCTE)^10,11^; when compared to VCTE, MRI-based techniques are more expensive and time consuming to implement. Therefore, it is clinically useful to know the difference in diagnostic performance between these two techniques.

In this study, we investigated the accuracy of VCTE and magnetic resonance elastography (MRE) to detect liver fibrosis and the accuracy of controlled attenuation parameter (CAP) measurements. Similarly, we performed and MRI-based proton density fat fraction (MRI-PDFF) to detect liver steatosis among patients with overweight and obese.

## Methods

### Study design

We conducted a cross-sectional retrospective study of patients with NAFLD who had undergone all three examinations (liver biopsy, VCTE and MRE) within 6 months between January 2014 and March 2020 at Yokohama City University Hospital. The study protocol complied with the ethical principles of the 1975 Declaration of Helsinki and was approved by the Ethics Committee of Yokohama City University Hospital (Yokohama, Japan; approval number B2104, April 28, 2021), and all patients provided written informed consent. The inclusion and exclusion criteria are described in Supplementary Online Resource 1.

### Definition of overweight and obese

According to the World Health Organization definition, obesity is defined as a body mass index (BMI) ≥ 30 kg/m^2^, and overweight is defined as a BMI > 25 and < 30 kg/m^2^^12^. Notably, Asians have high prevalence of type 2 diabetes and cardiovascular risk factors, even among normal weight populations (those with a BMI > 25 kg/m^2^)^13^. In 2004, the World Health Organization Western Pacific Regional Office established a different definition of obesity for Asians, where the BMI was categorized as “underweight” (< 18.4 kg/m^2^), “normal” (≥ 18.5–22.9 kg/m^2^), “overweight at risk (overweight)” (≥ 23.0–24.9 kg/m^2^), “obese I” (≥ 25.0–29.9 kg/m^2^), and “obese II” (≥ 30.0 kg/m^2^)^13^. In our country, the Japanese Society for the Study of Obesity defines obesity as a BMI of 25 > kg/m^2^ or above^14^. In the current study, we used international standards to define BMI < 25 as “normal,” BMI > 25 and < 30 kg/m^2^ as “overweight,” and BMI ≥ 30 kg/m^2^ as “obese.”

### Histological findings

Liver biopsy was performed in all participants. The procedure and methods are described in Supplementary Online Resource 2. Grading and staging were based on the NASH clinical network criteria, as previously reported. Steatosis affecting < 5%, 5%–33%, 33–66% and > 66% of hepatocytes was classified as grade 0, 1, 2 and 3, respectively. Lobular inflammation was graded according to the number of inflammatory foci per field of view at a magnification of 200× , with 0, < 2, 2 − 4, and > 4 foci per field classified as grades 0, 1, 2, and 3, respectively. Hepatocellular ballooning involving no, few, and many cells was classified as grade 0, 1, and 2, respectively^15^.

### Vibration controlled transient elastography

VCTE data were determined utilizing the M or XL probes of FibroScan (EchoSens, Paris, France). The probe to liver capsule distance (PCD), defined as the layer between the skin and the liver capsule, was measured to determine which probe size should be used. The M probe was used when the PCD was < 25 mm, and the XL probe was used when the PCD was > 25 mm. The details of the methods and testing procedures have been reported previously^16,17^. The patient was placed in a supine position with the right arm raised to the maximum height, and the liver stiffness measurement (LSM) of the right lobe of the liver was measured from the intercostal space. The LSM value was calculated as the median value and expressed in kilopascals (kPa).

The CAP values provided by the device were used for evaluation only if the VCTE was valid for the same signal. Thus, CAP values were simultaneously measured from the same volume of liver parenchyma as the VCTE.

Reliable VCTE were defined as those with a success rate (ratio of number of acquisitions) of at least 60% and a median interquartile range (interquartile range = range of the middle 50% of the data) value of < 30% among 10 valid measurements. Unreliable VCTE were defined as fewer than 10 valid acquisitions, a success rate < 60%, and/or an interquartile range/median ≥ 30%, as previously reported^16,17^.

### MRI proton density fat fraction and MR elastography

Liver MRE was performed utilizing 3.0 Tesla imagers (GE Healthcare, Milwaukee, WI) at our institution according to previously described methods^18^. MRI-PDFF was measured by a modified Dixon method with advanced processing (IDEAL IQ, GE Healthcare), utilizing the method previously published^19–21^. A region of interest was drawn to measure MRE, including only the parenchyma of the right lobe and avoiding the liver edge and surface, large vessels, bile ducts, gallbladder, tumor, and artifacts. The average of the measurements from four slices was used for the analysis. Examinations were considered adequate if the total number of pixels over four slices acquired in a participant was greater than or equal to 700 pixels^22^. To measure MRI-PDFF, another region of interest was drawn on the in- and out-of-phase images near the site where the region of interest was drawn for MRE.

**Figure 1:**
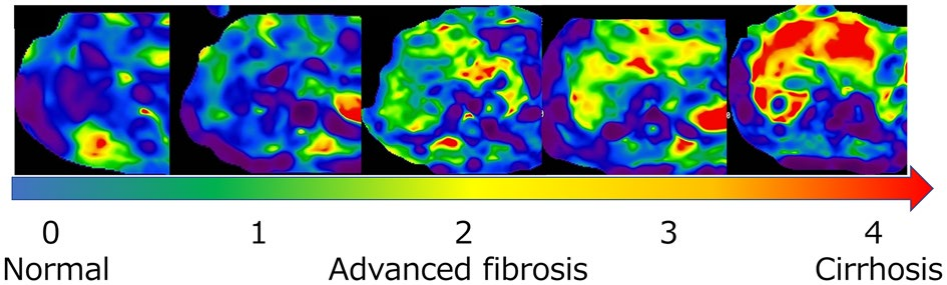
MRE colorized images. *MRE* magnetic resonance elastography.

MRE and MRI-PDFF obtained simultaneously were registered in a database, extracted for this study and analyzed by one of the authors who was blinded to the pathological results (K. I.). With the equipment used in this study, MRI-PDFF is a black-and-white image, thus, making it difficult to visually assess liver steatosis. However, MRE can also be colorized and displayed, allowing visual assessment of LSM (Fig. 1).

### Clinical features, laboratory characteristics and scoring systems

Detailed histories, physical measurements, and biochemical tests were obtained from all participants. Each patient’s height and weight were measured using a certified scale after removal of shoes and any heavy clothing. The BMI was calculated as weight (kg)/height (m)^2^. After a night of fasting (12 h), a venous blood draw was performed, and the following parameters were measured: platelet count (PC), albumin level, aspartate aminotransferase (AST) level, alanine aminotransferase (ALT) level, type IV collagen 7 s, hyaluronic acid, fasting blood glucose level, and glycosylated hemoglobin level. Standard methods were used to measure these parameters.

The ratio formula of Fibrosis-4 index^23^, AST to ALT ratio^24^, AST to platelet ratio index^25^, and NAFLD fibrosis score^26^ are described in the supplementary material.

### Statistical analysis

Statistical analysis was conducted by using JMP statistics software (version 15.0.0; SAS Institute, Cary, NC, USA). Univariate comparisons between patient groups were performed with the use of Student’s *t* test. The 95% CIs were calculated using the Woolf method. Jackknife tests were used to compare the area under the receiver operating curve (AUROC) between the two groups. Statistical significance was set at P < 0.05.

## Results

### Patient characteristics

**Figure 2:**
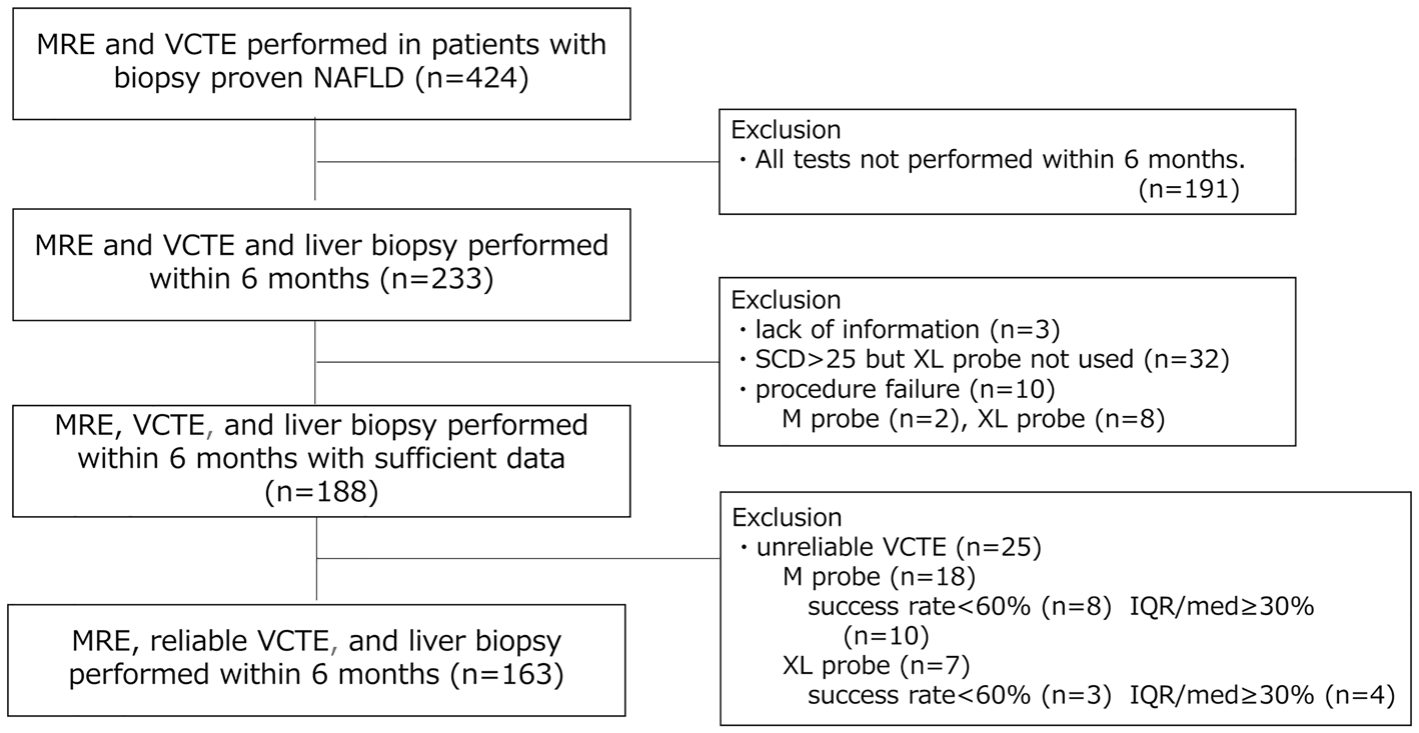
Flow chart of patient enrollment.

Between January 2014 and March 2020, 424 patients underwent liver biopsy, VCTE, and MRE at our center. Patients who did not undergo all three tests within 6 months were excluded (n = 191). Patients with insufficient information (n = 3), and those with PCD ≥ 25 mm in whom an XL probe was not used (n = 32), procedure failure (n = 10), or unreliable VCTE (n = 25) were also excluded. Finally, the data of 163 patients were analyzed. A flowchart of patient enrolment is shown in Fig. 2. The fibrosis stage and steatosis grade were evaluated in all patients based on the liver biopsy (Table 1). The number of patients with fibrosis stage 0, ≥ 1 ≥ , ≥ 2, ≥ 3 and 4 assessed by liver biopsy was 2, 15, 18, 5, 13 and 0 for those with BMI < 25 kg/m^2^; 1, 10, 21, 25 and 11 for those with BMI ≥ 25 and < 30 kg/m^2^; and 1, 8, 11, 23 and 14 for those with BMI ≥ 30 kg/m^2^, respectively. None of the patients with a BMI > 25 kg/m^2^ had stage 4 fibrosis. The numbers of patients with steatosis grades 0, ≥ 1, ≥ 2 and 3 assessed by liver biopsy with BMI < 25, ≥ 25 and < 30 and ≥ 30 kg/m^2^ were 4, 18, 12 and 4; 2, 33, 21, and 12; and 1, 22, 21 and 13, respectively. Analyses were conducted according to three groups stratified by BMI: normal (BMI < 25 kg/m^2^, n = 38), overweight (BMI > 25 and < 30 kg/m^2^, n = 68), and obese (BMI ≥ 30 kg/m^2^, n = 57). Patient characteristics and laboratory findings for the entire study population and by group are presented in Table 1. The M probe was used when the PCD was < 25 mm and the XL probe was used when the PCD was > 25 mm; as BMI increased, the PCD tended to increase, and the XL probe was used in more patients.

**Table 1:** Patient characteristics.

Characteristics	All (n = 163)	BMI < 25 (n = 38)	25 ≤ BMI < 30 (n = 68)	BMI ≥ 30 (n = 57)
Age, years	59.7 ± 13.0	63.8 ± 12.0	59.5 ± 13.7	57.1 ± 12.4
Sex, male (%)	86 (52.8)	17 (44.7)	38 (55.9)	31 (54.4)
Height, m	1.6 ± 0.1	1.6 ± 0.0	1.6 ± 0.1	1.6 ± 0.1
Body weight, kg	75.1 ± 15.5	59.7 ± 8.2	72.6 ± 9.2	88.5 ± 14.3
BMI, kg/m^2^	28.5 ± 4.5	23.0 ± 1.6	27.4 ± 1.4	33.4 ± 3.0
PCD mm	22.5 ± 4.9	19.3 ± 3.8	21.8 ± 4.0	25.4 ± 44.2
M probe: XL probe	131:32	37:1	60:8	34:23
VCTE-LSM, kPa	11.9 ± 7.7	9.8 ± 5.2	11.9 ± 9.1	13.3 ± 6.9
CAP, using VCTE	289.4 ± 47.6	259.5 ± 42.8	285.9 ± 42.6	312.8 ± 44.2
MRE, kPa	4.3 ± 7.6	3.9 ± 1.4	4.4 ± 1.6	4.6 ± 1.5
MRI-PDFF, %	11.7 ± 7.0	9.8 ± 8.2	12.2 ± 6.9	12.3 ± 6.0
AST, IU/L	52.1 ± 27.5	52.8 ± 34.0	53.2 ± 27.7	50.3 ± 22.2
ALT, IU/L	65.4 ± 41.1	64.7 ± 42.5	65.5 ± 43.7	65.8 ± 37.7
GGT, IU/L	93.1 ± 83.9	93.7 ± 79.1	102.2 ± 98.8	81.8 ± 65.7
Platelet count	19.2 ± 7.0	18.3 ± 6.3	19.9 ± 7.0	19.0 ± 7.5
Type IV collagen 7 s	6.5 ± 2.3	6.0 ± 1.9	6.8 ± 2.7	6.6 ± 1.9
Hyaluronic acid	87.1 ± 80.7	87.9 ± 1.9	86.4 ± 76.5	87.5 ± 70.6
AAR	2.7 ± 2.5	0.9 ± 2.9	1.0 ± 0.5	0.9 ± 0.3
APRI	0.9 ± 0.4	1.3 ± 1.1	1.1 ± 0.7	1.2 ± 0.6
FIB-4 index	2.7 ± 2.5	3.1 ± 2.9	2.8 ± 2.9	2.5 ± 1.7
NFS	-0.5 ± 1.8	-1.0 ± 1.7	-0.7 ± 1.9	0.1 ± 1.8
Hypertension, n (%)	86 (52.8)	13 (34.2)	34 (50.0)	39 (68.4)
Diabetes mellitus, n (%)	100 (61.3)	17 (44.7)	42 (61.8)	41 (71.9)
Dyslipidemia, n (%)	116 (71.2)	23 (60.5)	46 (67.6)	47 (82.5)
**Steatosis grade, n (%)**				
< 5%	7 (4.3)	4 (10.5)	2 (2.9)	1 (1.7)
5–33%	73 (44.8)	18 (47.4)	33 (48.5)	22 (38.6)
33–66%	54 (33.1)	12 (31.6)	21 (30.9)	21 (36.2)
> 66%	29 (17.8)	4 (10.5)	12 (17.6)	13 (22.8)
Steatosis grade (biopsy)	1.6 ± 0.8	1.4 ± 0.8	1.6 ± 0.8	1.8 ± 0.8
Steatosis grade 0, n (%)	7	4	2	1
Steatosis grade 1, n (%)	73	18	33	22
Steatosis grade 2, n (%)	54	12	21	21
Steatosis grade 3, n (%)	29	4	12	13
**Lobular inflammation grade, n (%)**				
None	4 (2.5)	1 (2.6)	1 (1.7)	2 (3.5)
< 2 foci per × 200 field	92 (56.4)	26 (68.4)	37 (54.4)	9 (50.9)
2–4 foci per × 200 field	61 (37.4)	11 (28.9)	25 (36.8)	25 (43.9)
> 4 foci per × 200 field	6 (3.7)	0 (0.0)	5 (7.4)	1 (1.7)
**Liver cell ballooning grade, n (%)**				
None	49 (30.1)	19 (50.0)	17 (25.0)	13 (22.8)
Few balloon cells	92 (56.4)	16 (42.1)	42 (61.8)	34 (59.6)
Many balloon cells	22 (13.5)	3 (7.9)	9 (13.2)	10 (17.5)
**Fibrosis stage, n (%)**				
None	4 (2.5)	2 (5.3)	1 (1.5)	1 (1.8)
Perisinusoidal or periportal	36 (22.1)	18 (47.4)	10 (14.7)	8 (14.9)
Perisinusoidal and portal/periportal	37 (22.7)	5 (13.2)	21 (30.9)	11 (19.3)
Bridging fibrosis	61 (37.4)	13 (34.2)	25 (36.8)	23 (40.4)
Cirrhosis	25 (15.3)	0 (0.0)	11 (16.2)	14 (24.6)
Fibrosis stage (biopsy)	2.4 ± 1.1	1.8 ± 1.0	2.5 ± 1.0	2.7 ± 1.1
Fibrosis stage 0, n (%)	4	2	1	1
Fibrosis stage 1, n (%)	36	18	10	8
Fibrosis stage 2, n (%)	37	5	21	11
Fibrosis stage 3, n (%)	61	13	25	23
Fibrosis stage 4, n (%)	25	0	11	14

Data are presented as means ± standard deviations.

*AAR* AST to ALT ratio, *ALT* alanine aminotransferase, *APRI* AST to platelet ratio index, *AST* aspartate aminotransferase, *BMI* body mass index, *CAP* controlled attenuation parameter, *FIB-4* fibrosis 4, *GGT* γ-glutamyl transpeptidase, *LSM* liver stiffness measurement, *MRE* magnetic resonance elastography, *MRI* magnetic resonance imaging, *MRI-PDFF* magnetic resonance imaging-proton density fat fraction, *NAFLD* nonalcoholic fatty liver disease, *NAS* NAFLD activity score, *NFS* NAFLD fibrosis score, *PCD* probe-to-liver capsule distance, *NFS* non-alcoholic fatty liver disease fibrosis score, *VCTE* vibration-controlled transient elastography.

### Analysis of liver fibrosis in groups according to BMI

**Figure 3:**
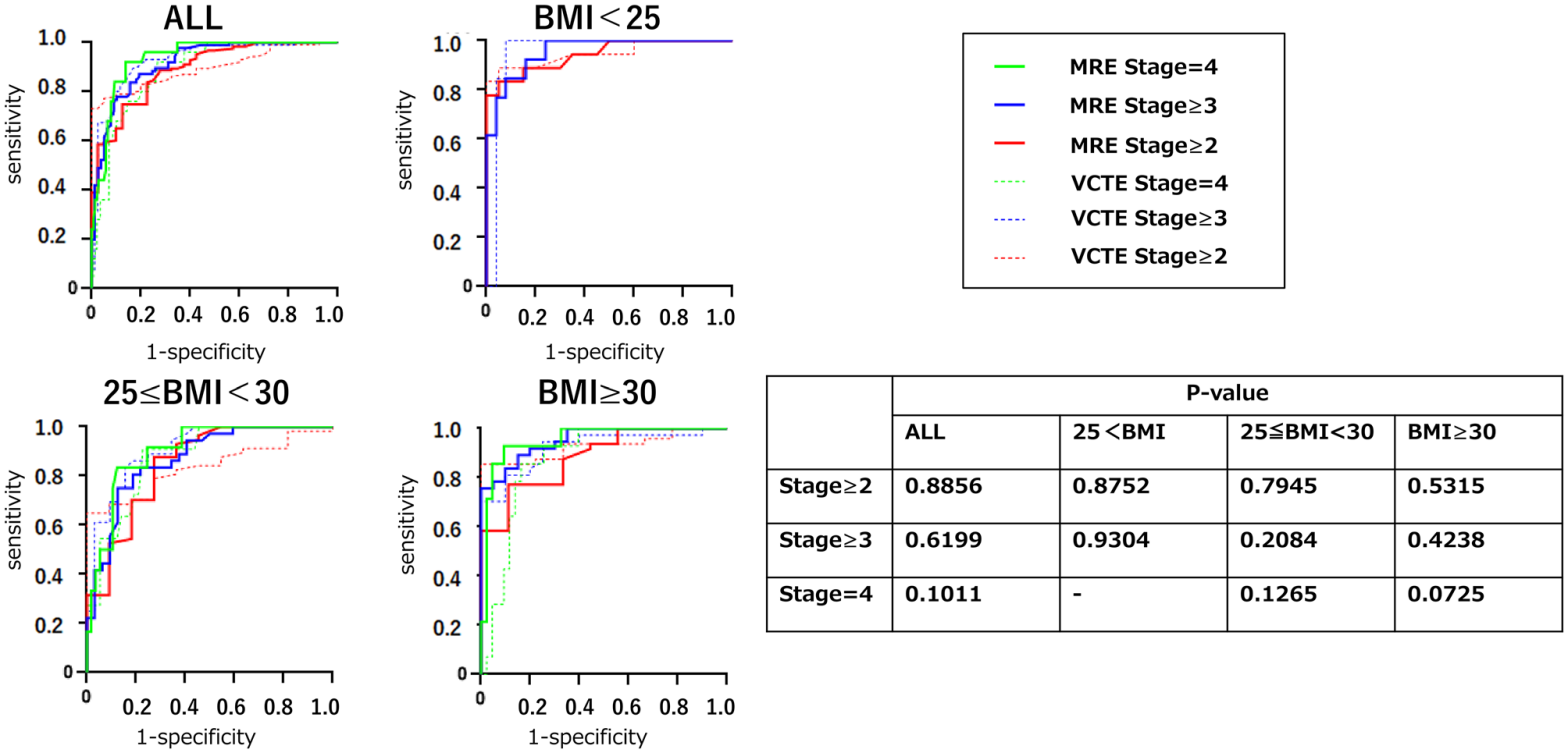
Specific cut-offs to define each grade of steatosis and stage of fibrosis by VCTE and MRI/MRE techniques. *MRE* magnetic resonance elastography, *MRI* magnetic resonance imaging, *VCTE* vibration-controlled transient elastography.

**Figure 4:**
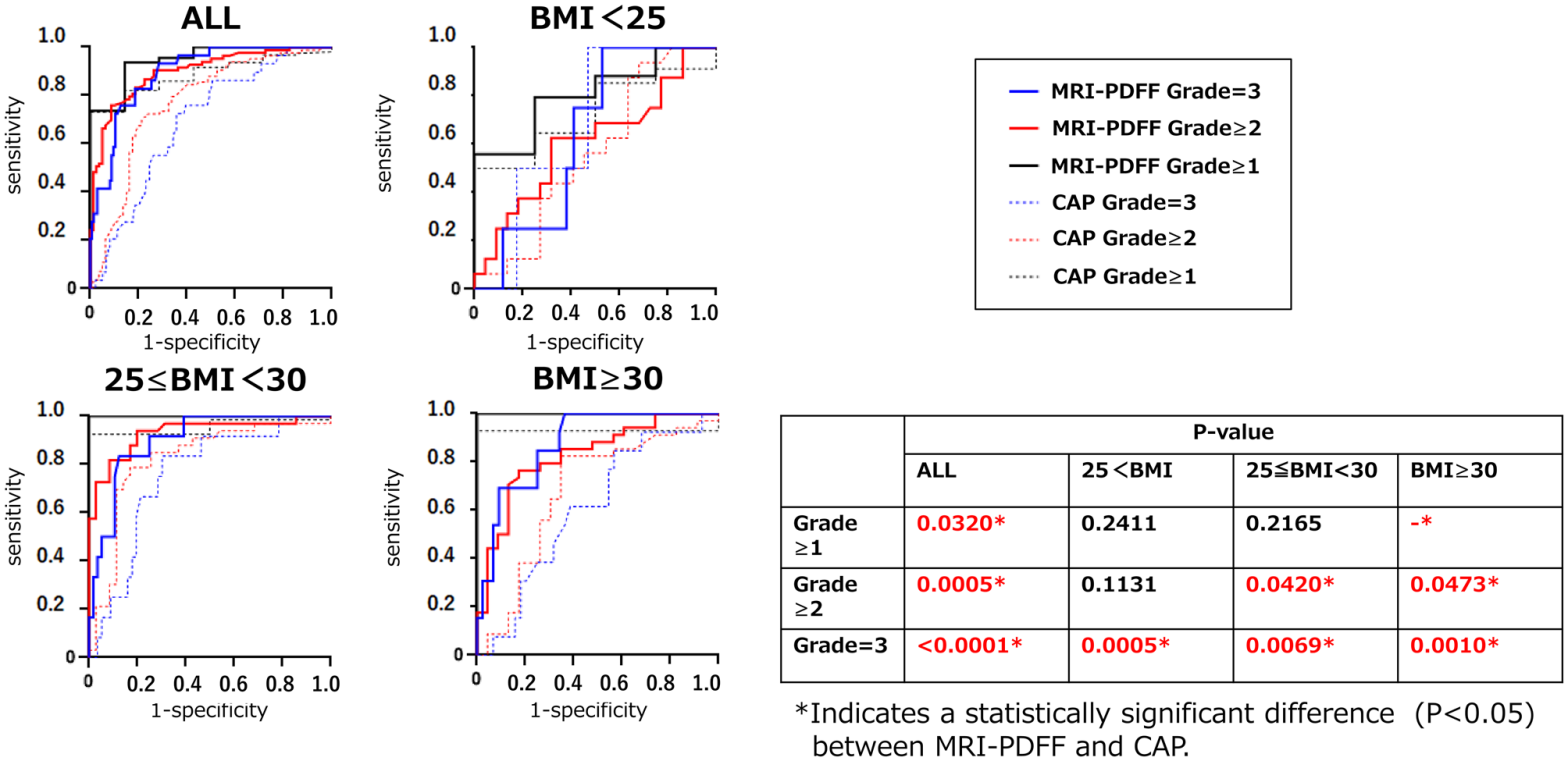
Comparison of the diagnostic accuracy of VCTE and MRI/MRE techniques to detect liver fibrosis in patients with NAFLD presented by AUROCs. *MRE* magnetic resonance elastography, *MRI* magnetic resonance imaging, *VCTE* vibration-controlled transient elastography, *AUROC* area under the receiver operating curve.

We examined the diagnostic accuracy of LSM measured by VCTE and MRE, by comparing the AUROCs. The analysis was performed according to the BMI groups specified above. Jackknife tests were performed to investigate if there were significant differences in the results between the groups according to BMI (Table 2). VCTE and MRE predicted liver fibrosis of stage 2 or above in patients with NAFLD with an AUROC of 0.89/0.94/0.85/0.88 (MRE) and 0.90/0.95/0.83/0.94 (VCTE) for overall/normal/overweight/obese participants, respectively. MRE predicted liver fibrosis of stage 3 or above in patients with NAFLD with an AUROC of 0.92/0.96/0.87/0.95 (MRE) and 0.93/0.95/0.91/0.92 (VCTE) for overall/normal/overweight/obese, respectively. VCTE and MRE predicted liver fibrosis 4 (cirrhosis) in patients with NAFLD with an AUROC of 0.94/-/0.95/0.95 (MRE) and 0.89/-/0.88/0.87 (VCTE) for overall/normal/overweight/obese patients, respectively. The specific cut-offs used in this study are presented in Fig. 3. There was no considerable difference in the AUROCs according to the BMI in all groups (Fig. 4).

**Table 2:** Diagnostic ability of VCTE and MRI/MRE techniques to detect hepatic fibrosis and steatosis presented by AUROCs in patients with NAFLD.

		Fibrosis stage		
		F0–1 vs. F2–4	F0–2 vs. F3–4	F0–3 vs. F4
All	MRE	0.89	0.92	0.94
	VCTE	0.90	0.93	0.89
BMI < 25 kg/m^2^ normal	MRE	0.94	0.96	–
	VCTE	0.95	0.95	–
25≦BMI < 30 kg/m^2^ overweight	MRE	0.85	0.87	0.95
	VCTE	0.83	0.91	0.88
BMI ≥ 30 kg/m^2^ obese	MRE	0.88	0.95	0.95
	VCTE	0.94	0.92	0.87

		Steatosis grade		
		S0 vs. S1–3	S0–1 vs. S2–3	S0–2 vs. S3
All	MRI-PDFF	0.95	0.90	0.89
	CAP	0.89	0.77	0.69
BMI < 25 kg/m^2^ normal	MRI-PDFF	0.81	0.93	0.90
	CAP	0.73	0.81	0.63
25≦BMI < 30 kg/m^2^ overweight	MRI-PDFF	1.00	0.93	0.90
	CAP	0.95	0.82	0.75
BMI ≥ 30 kg/m^2^ obese	MRI-PDFF	1.00	0.83	0.88
	CAP	0.93	0.68	0.61

*CAP* controlled attenuation parameter, *MRE* magnetic resonance elastography, *MRI-PDFF* magnetic resonance imaging-proton density fat fraction, *VCTE* vibration-controlled transient elastography.

### Analysis of liver steatosis measurements categorized by BMI

We examined the diagnostic accuracy of liver steatosis measured by CAP and MRI-PDFF among the different BMI categories by comparing their AUROCs (Table 2). CAP and MRI-PDFF detected liver steatosis of grade 1 or above in patients with NAFLD with an AUROC of 0.95/0.81/1.00/1.00 (MRI-PDFF) and 0.89/0.73/0.95/0.93 (CAP) for overall/normal/overweight/obese participants, respectively. MRI-PDFF and CAP predicted NAFLD with an AUROC of 0.90/0.93/0.93/0.83 (MRI-PDFF) and 0.77/0.81/0.82/0.68 (CAP) for overall/normal/overweight/obese participants, respectively. CAP and MRI-PDFF predicted liver steatosis of grade 3 or above in patients with NAFLD with an AUROC of 0.89/0.90/0.90/0.88 (MRI-PDFF) and 0.69/0.63/0.75/0.61 (CAP) for overall/normal/overweight/obese patients, respectively. The specific cut-offs used in this study are presented in Fig. 3.

**Figure 5:**
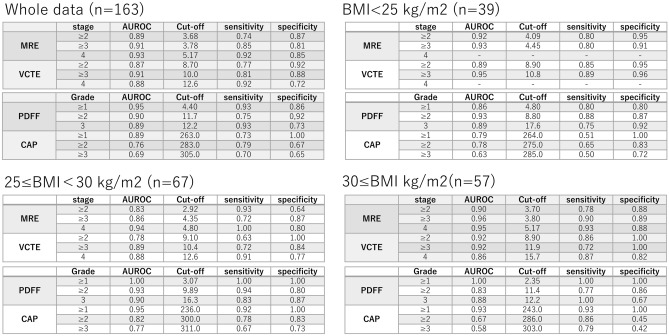
Comparison of the diagnostic accuracy of VCTE and MRI/MRE techniques to detect liver steatosis in patients with NAFLD presented by AUROCs. *MRE* magnetic resonance elastography, *MRI* magnetic resonance imaging, *VCTE* vibration-controlled transient elastography, *AUROC* area under the receiver operating curve.

Comparing the results of the AUROCs using the Jackknife test, there was a considerable difference in the assessment of liver steatosis in the total group, normal ≥ 1 and 2, and overweight ≥ 1. As the BMI increased, the difference between the CAP and MRI-PDFF became more considerable (Fig. 5).

### Factors influencing the success rate of VCTE

As shown in Fig. 2, 25 cases of unreliable VCTE were observed. There were no considerable differences in sex, age, height, weight, BMI, PCD, LSM and MRI-PDFF in the histological assessment of liver fibrosis and steatosis between reliable and unreliable VCTEs (Table 3). Among the patients with sufficient data who received liver biopsy, VCTE, and MRE within the 6-month time frame described above (n = 188), the percentage of patients with a reliable VCTE increased each year between 2014 and 2020. The percentage of patients undergoing VCTE increased annually. The rates are summarized in Supplementary Online Resource 3.

**Table 3:** Factors that may influence the implementation of reliable VCTE.

	Reliable examinations (n = 163)	Unreliable examinations (n = 25)	P-value
Age, years	59.7 ± 13.0	60.6 ± 13.7	0.75
Sex, male:female	86:77	10:15	0.24
Height, m	1.6 ± 0.10	1.60 ± 0.1	0.38
Body weight, kg	75.1 ± 15.5	71.90 ± 13.3	0.32
BMI, kg/m^2^	28.5 ± 4.5	28.0 ± 5.4	0.67
PCD mm	22.5 ± 4.9	22.9 ± 5.2	0.71
LSM, utilizing MRE	11.9 ± 7.7	4.4 ± 1.6	0.88
MRI-PDFF, utilizing MRI	11.7 ± 7.0	10.2 ± 4.4	0.28
Fibrosis stage	2.4 ± 1.1	2.2 ± 1.0	0.44
Steatosis grade	1.6 ± 0.8	1.5 ± 0.7	0.48

Data are presented as means ± standard deviations.

*BMI* body mass index, *LSM* liver stiffness measurement, *MRE* magnetic resonance elastography, *MRI* magnetic resonance imaging, *MRI-PDFF* magnetic resonance imaging-proton density fat fraction, *PCD* probe to liver capsule distance.

*P < 0.05.

## Discussion

Obesity is rising worldwide, and as the number of obese patients increases, so does the number of patients with NAFLD. It is anticipated that the diagnosis of NAFLD using non-invasive elastography will replace invasive diagnostic techniques, such as liver biopsy. Therefore, the diagnostic accuracy of elastography needs to be fully evaluated.

Asians are less likely to be obese than Caucasians but have a higher body fat percentage and higher risk of death from cardiovascular and other diseases despite lower BMIs^27^; therefore, the World Health Organisation has set criteria for obesity for Asians that differ from those for Caucasians^13^. It is imperative to correctly assess the extent of liver fibrosis not only in very obese people, but also in overweight and mildly obese people.

The choice of VCTE probes depends on the PCD. In a study of severely obese patients with an average BMI of 40 kg/m^2^, high diagnostic accuracy was achieved with proper use of the M and XL probes^28^. The present study was conducted in a population with a mean BMI of 28 kg/m^2^. The XL probe was used in one of 38 (2.6%) participants with normal BMI, eight out of 68 (11.8%) overweight participants, and 23 of 57 (40.4%) obese participants. PCD tended to increase with increasing BMI. Even in the overweight group (mean BMI, 27.35 kg/m^2^), the XL probe was used in 10% of patients, which is consistent with the findings of a report on the usefulness of the XL probe in overweight and obese patients^29^. In this group, PCD > 25 mm was found in 8% of patients with a BMI of 28–30 kg/m^2^, which was consistent with previous reports. In contrast, it has been reported that MRE can evaluate liver fibrosis with high diagnostic performance without being affected by the BMI or the degree of liver inflammation in the evaluation of NAFLD^30^. Although the degree of liver inflammation was not considered in our study, the MRE had a high diagnostic performance in assessing liver fibrosis by the BMI, which was previously reported.

In the assessment of liver fibrosis, both the VCTE and MRE could predict fibrosis of stage ≥ 2, ≥ 3, and 4 (cirrhosis) in patients with NAFLD with an AUROC ≥ 0.83 in all BMI groups. There were no considerable differences between the VCTE and MRE results. Previous reports have shown that both VCTE and MRE can assess liver fibrosis accurately with high diagnostic performance^10,31,32^, and it was reported that MRE is more accurate in identifying and staging liver fibrosis than VCTE^10,11^. In this study, VCTE was as highly diagnostic as MRE in the evaluation of LSM, with no difference in diagnostic performance, but MRE had a higher AUROC, as previously reported. It is possible that the background for the good VCTE results in this study was the relatively low BMI values, which made the VCTE measurement easier to perform.

Conversely, in the assessment of liver steatosis, MRI-PDFF could detect liver steatosis of grades ≥ 2 and ≥ 3 in patients with NAFLD with a good AUROC across all BMI groups, while the diagnostic ability of CAP was lower than that of MRI-PDFF across all BMI groups. Further, compared to MRI-PDFF, the diagnostic performance of CAP for the diagnosis of liver steatosis tended to decrease with increasing BMI. Especially, MRI-PDFF provided an excellent assessment of liver steatosis, but the CAP results were inferior to those of MRI-PDFF. Recently, Beyer et al. compared MRI- and ultrasound-derived indices of liver steatosis in a pooled cohort of 580 patients with NAFLD (mean age, 56 years; sex, 60% women; mean BMI, 31.39 [26.8–36.8 kg/m^2^])^33^. Their study assessed liver steatosis in the largest number of patients to date and concluded that MRI-PDFF could accurately diagnose individuals with the range of histological steatosis, and that CAP is suitable for identifying those with lower levels of fat (steatosis of grade 1 or above). In our study, the AUROCs of MRI-PDFF were higher than those of CAP. Our results indicated that CAP tended to be effective in identifying lower grade liver steatosis, specifically steatosis grade 1, and these are consistent with the findings reported by Beyer et al.^33^.

Regarding the examination success rate, this study found that the success rate of VCTE was 85.3%, and the success rate of MRE was 100%. Chen et al.^34^ reported that high BMI, increased chest circumference, and increased waist circumference were associated with unsuccessful VCTE, but neither PCD nor the type of VCTE probe was a considerable risk factor for unsuccessful VCTE examination. In our study, chest and waist circumference were not measured, but high BMI, PCD, and the type of VCTE probe were not risk factors for unsuccessful VCTE (Table 3). Further, Chen et al.^34^ reported that training or technical improvements improved VCTE AUROCs. Thus, we examined the years in which the tests were carried out and calculated the success rates for the investigations. The success rate tended to increase with each passing year, indicating that the performance of the machines and the skill of the examiner improved; this supported the postulation of Chen et al. that the AUROC of VCTE would increase with the number of studies performed by the examiners and the use of newer machines with better performance.

Several factors affecting the success rate of MRE have been identified, including claustrophobia, obesity to the extent that the patient cannot fit in the magnet bore, and hemochromatosis^35,36^. As this study did not include patients with claustrophobia or hemochromatosis, this likely contributed to the high MRE success rate.

There were some limitations to this study. First, the proportion of patients with advanced liver fibrosis in this population was small. Second, we cannot rule out referral bias and patient selection bias, as liver biopsy and elastography might have been performed more selectively in patients with NAFLD who may have already progressed to NASH. Therefore, the results of this study may not be representative of all patients with NAFLD. Third, this study was conducted in a single institution with a relatively small-sized sample and was assessed by a pathologist in a single institution. Fourth, our study is a retrospective analysis of cases, in which all three tests, MRE, VCTE, and liver biopsy, could be performed in a short period of time. In clinical practice, these investigations are not always accessible, and results may vary if they are performed prospectively. Fourth, previous studies have found a strong correlation between liver steatosis and viscosity by MRE^37,38^, but we have not discussed this issue. Viscosity is measured by measuring MRE at several different frequencies and finding the difference between them. In this retrospective study, viscosity was not evaluated because MRE was not measured at multiple frequencies. In addition, viscosity cannot be measured with the FibroScan used in this study. Therefore, evaluation of viscosity by BMI using noninvasive testing methods is an issue for future study.

In conclusion, this is the first report focusing on the diagnosis of liver fibrosis and steatosis using non-invasive techniques in overweight patients with NAFLD. Compared to other ethnic groups, Asians with a lower BMI are more likely to have NAFLD, and this research shows the diagnostic accuracy of non-invasive examinations in patients with average Asian body compositions. The key prognostic factor in NAFLD is the development of liver fibrosis^5–7^, and elastography, a non-invasive test, has been reported to be useful in maintaining long-term patient follow-up^39^. This study examines the diagnostic performance of VCTE and MRI techniques for assessing liver fibrosis and steatosis in overweight and obese patients with NAFLD, concluding that both are likely to be valuable for long-term follow-up.

In summary, both VCTE and MRE provide comparably good assessment of liver fibrosis, although the MRI-PDFF methods have a greater diagnostic ability for liver steatosis than CAP in overweight and obese patients with NAFLD.

## Supplementary Information

Supplementary Information 1.

Supplementary Information 2.

Supplementary Information 3.

## Data Availability

The datasets generated during and/or analyzed during the current study are available on supplementary file.

## References

[1] Younossi Z (2018). Global burden of NAFLD and NASH: Trends, predictions, risk factors and prevention. Nat. Rev. Gastroenterol. Hepatol..

[2] Harrison SA, Torgerson S, Hayashi PH (2003). The natural history of nonalcoholic fatty liver disease: A clinical histopathological study. Am. J. Gastroenterol..

[3] Bellentani S (2000). Prevalence of and risk factors for hepatic steatosis in Northern Italy. Ann. Intern. Med..

[4] Caldwell SH, Crespo DM (2004). The spectrum expanded: Cryptogenic cirrhosis and the natural history of non-alcoholic fatty liver disease. J. Hepatol..

[5] Angulo P (2015). Liver fibrosis, but no other histologic features, is associated with long-term outcomes of patients with nonalcoholic fatty liver disease. Gastroenterology.

[6] Dulai PS (2017). Increased risk of mortality by fibrosis stage in nonalcoholic fatty liver disease: Systematic review and meta-analysis. Hepatology.

[7] Hagström H (2017). Fibrosis stage but not NASH predicts mortality and time to development of severe liver disease in biopsy-proven NAFLD. J. Hepatol..

[8] Younossi ZM, Koenig AB, Abdelatif D, Fazel Y, Henry L, Wymer M (2016). Global epidemiology of nonalcoholic fatty liver disease-Meta-analytic assessment of prevalence, incidence and outcomes. Hepatology.

[9] Nah BKY (2022). Histological changes in weight classes and the influence of NAFLD prevalence: A population analysis of 34,486 individuals. Int. J. Environ. Res. Public Health.

[10] Imajo K (2016). Magnetic resonance imaging more accurately classifies steatosis and fibrosis in patients with nonalcoholic fatty liver disease than transient elastography. Gastroenterology.

[11] Dulai PS, Sirlin CV, Loomba R (2016). MRI and MRE for non-invasive quantitative assessment of hepatic steatosis and fibrosis in NAFLD and NASH: Clinical trials to clinical practice. J. Hepatol..

[12] WHO Expert Committee on Physical Status (1995). Physical status: The use and interpretation of anthropometry. Report of a WHO Expert Committee. World Health Organ. Tech. Rep. Ser..

[13] WHO Expert Consultation (2004). Appropriate body-mass index for Asian populations and its implications for policy and intervention strategies. Lancet.

[14] Japan Society for the Study of Obesity (2016). Guidelines for the Management of Obesity Disease 2016.

[15] Kleiner DE (2005). Design and validation of a histological scoring system for nonalcoholic fatty liver disease. Hepatology.

[16] Yoneda M (2008). Noninvasive assessment of liver fibrosis by measurement of stiffness in patients with nonalcoholic fatty liver disease (NAFLD). Dig. Liver. Dis..

[17] Sandrin L, Tanter M, Gennisson JL, Catheline S, Fink M (2002). Shear elasticity probe for soft tissues with 1-D transient elastography. IEEE Trans. Ultrason. Ferroelectr. Freq. Control.

[18] Yin M (2007). Assessment of hepatic fibrosis with magnetic resonance elastography. Clin. Gastroenterol. Hepatol..

[19] Levenson H (1991). Fatty infiltration of the liver: Quantification with phase-contrast MR imaging at 1.5 T vs. biopsy. AJR Am. J. Roentgenol..

[20] Bernstein MA, King KF, Zhou XJ (2004). Handbook of MRI Pulse Sequences.

[21] Hussain HK (2005). Hepatic fat fraction: MR imaging for quantitative measurement and display–early experience. Radiology.

[22] Jayakumar S (2019). Longitudinal correlations between MRE, MRI-ODFF and liver histology in patients with non-alcoholic steatohepatitis: Analysis of data from phase II trial of selonsertib. J. Hepatol..

[23] Sterling RK (2006). Development of a simple noninvasive index to predict significant fibrosis in patients with HIV/HCV coinfection. Hepatology.

[24] Williams AL, Hoofnagle AL (1988). Ratio of serum aspartate to alanine aminotransferase in chronic hepatitis. Relationship to cirrhosis. Gastroenterology.

[25] Wai CT (2003). A simple noninvasive index can predict both significant fibrosis and cirrhosis in patients with chronic hepatitis C. Hepatology.

[26] Angulo P (2007). The NAFLD fibrosis score: A noninvasive system that identifies liver fibrosis in patients with NAFLD. Hepatology.

[27] WHO Consultation on Obesity (1999: Geneva, Switzerland), World Health Organization (2000). Obesity: Preventing and Managing the Global Endemic.

[28] de Lédinghen V, Vergniol J, Foucher J, El-Hajbi F, Merrouche W, Rigalleau V (2010). Feasibility of liver transient elastography with FibroScan using a new probe for obese patients. Liver Int..

[29] Myers RP (2012). Feasibility and diagnostic performance of the FibroScan XL probe for liver stiffness measurement in overweight and obese patients. Hepatology.

[30] Singh S (2016). Magnetic resonance elastography for staging liver fibrosis in non-alcoholic fatty liver disease: A diagnostic accuracy systematic review and individual participant data pooled analysis. Eur. Radiol..

[31] Park CC (2017). Magnetic resonance elastography vs. transient elastography in detection of fibrosis and noninvasive measurement of steatosis in patients with biopsy- proven nonalcoholic fatty liver disease. Gastroenterology.

[32] Imajo K (2022). Direct comparison of US and MR elastography for staging liver fibrosis in patients with nonalcoholic fatty liver disease. Clin. Gastroenterol. Hepatol..

[33] Beyer C (2021). Comparison between magnetic resonance and ultrasound-derived indicators of hepatic steatosis in a pooled NAFLD cohort. PLoS ONE.

[34] Chen J (2017). Diagnostic performance of MR elastography and vibration-controlled transient elastography in the detection of hepatic fibrosis in patients with severe to morbid obesity. Radiology.

[35] Huwart L (2008). Magnetic resonance elastography for the noninvasive staging of liver fibrosis. Gastroenterology.

[36] Yoon JH (2014). Hepatic fibrosis: Prospective comparison of MR elastography and US shear-wave elastography for evaluation. Radiology.

[37] Hudert CA (2019). Tomoelastography for the evaluation of pediatric nonalcohlic fatty liver disease. Investig. Radiol..

[38] Reiter R (2020). Diagnostic performance of tomoelastography of the liver and spleen for staging hepatic fibrosis. Eur. Radiol..

[39] Nogami A (2019). Assessment of 10-year changes in liver stiffness using vibration-controlled transient elastography in non-alcoholic fatty liver disease. Hepatol. Res..

