# Supplementary figures and images for: Diagnostic comparison of vibration-controlled transient elastography and MRI techniques in overweight and obese patients with NAFLD
Source: Sci Rep. 2022 Dec 19;12:21925. doi: 10.1038/s41598-022-25843-6 (PMC9763419; doi:10.1038/s41598-022-25843-6)

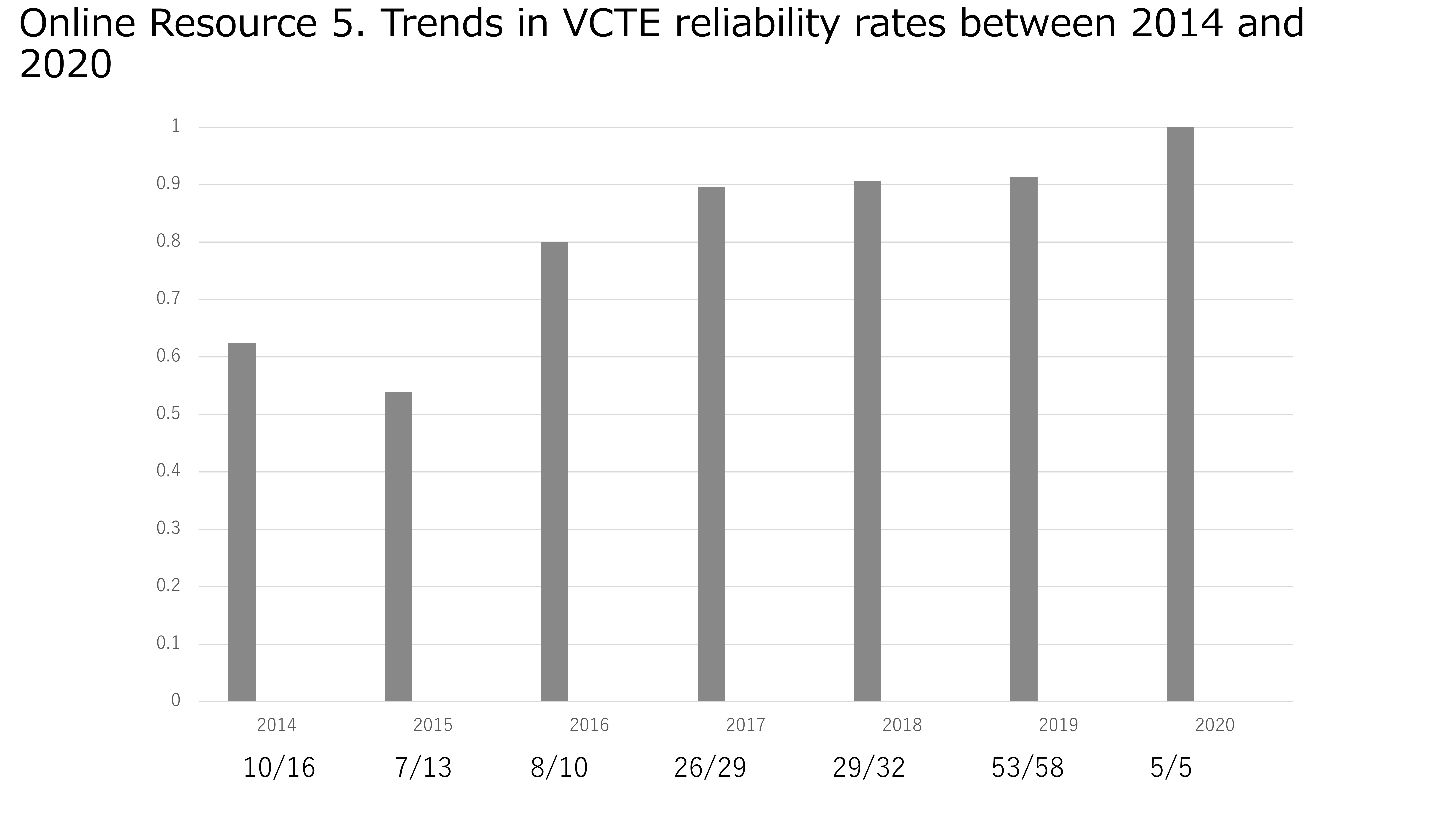

Supplement: Supplementary file 1 — Supplementary Information 1. [file 41598_2022_25843_MOESM1_ESM.tif]
