# Supplementary material for: Diagnostic comparison of vibration-controlled transient elastography and MRI techniques in overweight and obese patients with NAFLD
Source: Sci Rep. 2022 Dec 19;12:21925. doi: 10.1038/s41598-022-25843-6 (PMC9763419; doi:10.1038/s41598-022-25843-6)
Supplement: Supplementary file 3 — Supplementary Information 3. [file 41598_2022_25843_MOESM3_ESM.docx]

**Diagnostic Comparison of Vibration-controlled Transient Elastography and MRI techniques in Overweight and Obese NAFLD**

Asako Nogami^1^, Masato Yoneda^1 *^, Michihiro Iwaki^1^, Takashi Kobayashi^1^, Takaomi Kessoku^1^, Yasushi Honda^1^, Yuji Ogawa^2^, Kento Imajo^3^, Takuma Higurashi^1^, Kunihiro Hosono^1^, Hiroyuki Kirikoshi^4^, Satoru Saito^1^, Atsushi Nakajima^1^

*Correspondence: Masato Yoneda

Department of Gastroenterology and Hepatology, Yokohama City University Hospital, 3-9 Fukuura, Kanazawaku, Yokohama 236-0004, Japan

E-mail: yoneda-ycu@umin.ac.jp

Supplementary Online Resource 1. **Inclusion/exclusion criteria**

This is a retrospective analysis of adult patients with NAFLD who underwent liver biopsy at our institution for the purpose of the investigation of the causes of liver damage and participation in clinical trials or research. All patients were diagnosed with fatty liver disease, defined as the presence of steatosis in >5% of hepatocytes according to histological analysis. The exclusion criteria were daily alcohol intake of >30 g/day for men and >20 g/day for women, secondary causes of steatohepatitis, drug-induced liver injury, viral hepatitis, primary biliary cholangitis, autoimmune hepatitis, alpha-1-antitrypsin deficiency, hemochromatosis, Wilson's disease, and obstructive bile duct disease.

Supplementary Online Resource 2. **Histological findings**

Percutaneous liver biopsy was performed using a 16–18-gauge needle. Liver biopsy specimens were stained with hematoxylin–eosin and Masson’s trichrome stain. All liver biopsy specimens were subsequently analyzed by an experienced pathologist who was blinded to the VCTE, MRI, and clinical data. The histological diagnosis of NAFLD was based on the presence of hepatic steatosis deposits of 5% or more, as described by the American Association for the Study of Liver Diseases (AASLD) [1] and European Association for the Study of the Liver (EASL) [2].

Supplementary Onlice Resource 3. **Vibration Controlled Transient Elastography**

The patient was placed in a supine position with the right arm raised to the maximum height and the liver stiffness measurement (LSM) of the right lobe of the liver was measured from the intercostal space. The LSM was measured as the median value and expressed in kilopascals (kPa).

Supplementary Online Resource 4. **Scoring Systems**

The Fibrosis-4 (FIB-4) index was determined as age × AST (IU/L) / platelet count (×10^9^/L) / √ALT (IU/L). The AST to ALT ratio was calculated as AST/ALT. The AST to platelet ratio index was counted as AST (/ULN) / platelet count (×10^9^/L) × 100. The NAFLD fibrosis score was calculated using the following equation: −1.675 + 0.037 × age (years) + 0.094 × BMI (kg/m^2^) + 1.13 × impaired fasting glucose/diabetes mellitus (yes = 0, no = 1) + 0.99 × AST/ALT ratio – 0.013 × platelet count (×10^9^/L) − 0.66 × albumin (g/dL).

**References**

[1]. Chalasani, N. *et al.* The diagnosis and management of nonalcoholic fatty liver disease: practice guidance from the American Association for the Study of Liver Diseases*. Hepatology* **67,** 328-357 (2018).

[2]. European Association for the Study of the Liver (EASL), European Association for the Study of Diabetes (EASD) & European Association for the Study of Obesity (EASO). EASL-EASD-EASO Clinical Practice Guidelines for the management of non-alcoholic fatty liver disease*. J. Hepatol.* **64,** 1388-1402 (2016).
